# A risk stratification model based on four novel biomarkers predicts prognosis for patients with renal cell carcinoma

**DOI:** 10.1186/s12957-020-02046-9

**Published:** 2020-10-22

**Authors:** Shigehisa Kubota, Tetsuya Yoshida, Susumu Kageyama, Takahiro Isono, Takeshi Yuasa, Junji Yonese, Ryoji Kushima, Akihiro Kawauchi, Tokuhiro Chano

**Affiliations:** 1 Department of Urology, Shiga University of Medical Science, SetaTshukinowa-cho, Otsu, Shiga 520-2192 Japan; 2 Central Research Laboratory, Shiga University of Medical Science, SetaTshukinowa-cho, Otsu, Shiga 520-2192 Japan; 3 Department of Urology, Cancer Institute Hospital, Japanese Foundation for Cancer Research, Ariake, Koto, Tokyo, 135-8550 Japan; 4 Department of Clinical Laboratory Medicine, Shiga University of Medical Science, SetaTshukinowa-cho, Otsu, Shiga 520-2192 Japan; 5 Department of Medical Genetics, Shiga University of Medical Science, SetaTshukinowa-cho, Otsu, Shiga 520-2192 Japan

**Keywords:** Renal cell carcinoma (RCC), Global transcriptome analysis, Prognostic model, Multiple biomarkers, ADP-ribosylation factor-like 4C (ARL4C), Epithelial cell transforming 2 (ECT2), Superoxide dismutase 2 (SOD2), STEAP3 metalloreductase (STEAP3)

## Abstract

****Background**:**

Accurate prediction of the prognosis of RCC using a single biomarker is challenging due to the genetic heterogeneity of the disease. However, it is essential to develop an accurate system to allow better patient selection for optimal treatment strategies. ARL4C, ECT2, SOD2, and STEAP3 are novel molecular biomarkers identified in earlier studies as survival-related genes by comprehensive analyses of 43 primary RCC tissues and RCC cell lines.

****Methods**:**

To develop a prognostic model based on these multiple biomarkers, the expression of four biomarkers ARL4C, ECT2, SOD2, and STEAP3 in primary RCC tissue were semi-quantitatively investigated by immunohistochemical analysis in an independent cohort of 97 patients who underwent nephrectomy, and the clinical significance of these biomarkers were analyzed by survival analysis using Kaplan-Meier curves. The prognostic model was constructed by calculation of the contribution score to prognosis of each biomarker on Cox regression analysis, and its prognostic performance was validated.

****Results**:**

Patients whose tumors had high expression of the individual biomarkers had shorter cancer-specific survival (CSS) from the time of primary nephrectomy. The prognostic model based on four biomarkers segregated the patients into a high- and low-risk scored group according to defined cut-off value. This approach was more robust in predicting CSS compared to each single biomarker alone in the total of 97 patients with RCC. Especially in the 36 metastatic RCC patients, our prognostic model could more accurately predict early events within 2 years of diagnosis of metastasis. In addition, high risk-scored patients with particular strong SOD2 expression had a much worse prognosis in 25 patients with metastatic RCC who were treated with molecular targeting agents.

****Conclusions**:**

Our findings indicate that a prognostic model based on four novel biomarkers provides valuable data for prediction of clinical prognosis and useful information for considering the follow-up conditions and therapeutic strategies for patients with primary and metastatic RCC.

## Background

Renal cell carcinoma (RCC) represents 2–3% of all malignancies. It is the third most common malignant tumor among genitourinary tract cancers and its incidence is increasing [1]. Localized disease is commonly diagnosed as an incidental finding and can be successfully managed with nephrectomy, whereas metastatic RCC is refractory to conventional chemotherapy. Over the last decade, the availability of molecular targeted therapies which inhibit vascular endothelial cell growth factor receptor (VEGFR) and mammalian target of rapamycin (mTOR) kinase has led to a substantial improvement in outcome for patients with metastatic RCC [2]. However, curative treatment is still not available and the disease finally progresses, leading to the death of the patient. The clinical outcome for individual patients with metastatic RCC varies widely and predicting survival is challenging because of its genetic heterogeneity. Numerous molecular biomarkers including gene expression profiling and deep- and whole-genome sequencing have been investigated, but few single biomarkers offer predictability across datasets and clinical cohorts [3]. Hence, biomarkers and models that can predict prognosis and thus aid drug development for RCC are urgently needed. In some cancers, models based on the expression of multiple genes have been developed to predict survival and validated across datasets and study populations [4–7].

In previous work, we have demonstrated that RCC can be divided into two types in response to nutritional deprivation conditions, namely, starvation-resistant and -sensitive cells [8]. The former had higher activity of mitochondrial oxidative phosphorylation and glycolysis than the latter, and included a low level of mitochondrial reactive oxidative species due to sustained superoxide dismutase 2 (SOD2) contributing to survival under conditions of nutritional deprivation. This in turn contributed to the dormant character of RCC development. Higher SOD2 expression in primary RCC tissues was associated with significantly shorter survival in patients with metastatic RCC [9, 10]. To identify novel prognostic biomarkers of poor prognosis, we performed global transcriptome analysis on 43 primary RCC tissues by next generation sequencing and detected 29 coding genes which were highly expressed specifically in the worst prognosis groups. Seven survival-related genes (ARL4C, BIRC5, BUB1, CPS1, ECT2, FSTL1, and STEAP3), whose higher levels of mRNA expression were correlated with poorer prognosis, were extracted by receiver operating characteristic curve analysis with an accuracy of > 90% for predicting the prognosis of these 43 patients [11].

In the present study, we have collected the tissue-based biomarkers related to RCC patients, to validate whether these biomarkers can offer us the novelty for the therapeutic prognosis and for selection guidance of molecular targeting drugs. In addition, to promote clinical utility, the expression profile of individual biomarkers should be immunohistochemically evaluated using formalin-fixed paraffin-embedded tissue sections in an independent cohort of 97 RCC patients. In these respects, the present analysis has excluded BIRC5 and BUB1, which had been well-established prognostic function for RCC patients [12, 13]. In addition, CPS1 and FSTL1 could be hardly evaluated by IHC, for the tumor-specific expression. Therefore, the following biomarkers have been included in the present study: ADP-ribosylation factor-like 4C (ARL4C), epithelial cell transforming 2 (ECT2) and STEAP3 metalloreductase (STEAP3), and SOD2. The prognostic impacts were investigated using Kaplan-Meier curves, and the risk score of each biomarker for prognosis was calculated through Cox regression analysis. We developed an immunohistochemical staining-based prognostic model by integrating the expression patterns of individual biomarkers and their risk score. We aimed to validate whether our prognostic model can predict the prognosis more accurately than each biomarker separately and be available to guide targeted drug selection. The goal of this study is to select patients in need of particular treatments and to determine the optimal treatment strategy.

## Materials and methods

### Patients

Previously, higher levels of ARL4C, ECT2, SOD2, and STEAP3 mRNA in primary RCC tissues were reported to be associated with worse prognosis for patients with metastatic RCC [9, 11]. In the present study, we performed expression analysis by IHC for these four gene products and developed a risk model based on the expression profiles.

Ninety-seven patients who underwent radical or partial nephrectomy for the treatment of RCCs at the Shiga University of Medical Science Hospital from January 1999 to March 2016 were enrolled, and followed up to death or to October 2016. For this study, clinical and pathological data were obtained from medical records with written informed consent from individual patients and approval by the Ethics Committee of the institute (No. R2019-165). The median age of the 97 patients was 62 years (34–82 years). The histological subtypes of RCC were as follows: clear cell (*n* = 76, 78.4%), papillary (*n* = 12, 12.4%), chromophobe carcinomas (*n* = 8, 8.2%), and unclassified (*n* = 1, 1.0%). T stage was pT1 in 68 (70.1%), pT2 in 4 (4.1%), pT3 in 19 (19.6%), and pT4 in 6 (6.2%) patients. Vascular invasion and higher grading (G3) were identified in 67 (70.1%) and 18 (10.3%) patients, respectively. Histological diagnosis and pathological grading were according to the WHO Histological Classification system [14], while the clinical stage was according to the UICC TNM classification [15]. Surgery was performed in 70 (72.2%) patients as a curative measure, whereas the remaining 27 (27.8%) patients underwent cytoreductive nephrectomy because of the presence of distant metastasis at the time of RCC diagnosis.

During the follow-up period, 9 patients developed distant metastasis, and therefore total 36 cases were categorized as metastatic RCC patients. Finally, 16 fatalities were observed, and all of them were due to progression of RCC. The median follow-up period was 35 months (1–232 months) in this cohort.

Among 36 metastatic RCC patients, only 25 cases could be applied to the treatments of molecular targeting agents, due to the drug-approval status of the governmental administration in Japan. The 25 cases were treated with sorafenib (*n* = 4), sunitinib (*n* = 17), axitinib (*n* = 14), everolimus (*n* = 8), and pazopanib (*n* = 6). Maximum treatment responses were partial response (*n* = 10), stable disease (*n* = 13), progressive disease (*n* = 1), and not available (*n* = 1).

### Immunohistochemistry

Surgical specimens were transferred to 10% buffered formalin and fixed overnight. Fixed samples were embedded in paraffin and serially sliced into 5 μm sections. After dewaxing, sections were autoclaved at 120 °C for 1 min in 10 mM sodium citrate buffer (pH 6.0) and immersed in 0.3% H_2_O_2_. They were then incubated overnight at 4 °C with primary antibodies against ARL4C (diluted 1:400, #10202-1-AP, Proteintech, IL), ECT2 (diluted 1:200, #CF807408, Origene, MD), SOD2 (diluted 1:1000, #06-984, Millipore Corporation, Billerica, MA) and STEAP3 (diluted 1:400, #HPA050510, Atlas Antibodies, Stockholm, Sweden). Sections were rinsed with phosphate-buffered saline and incubated with secondary antibody conjugated with horseradish peroxidase (Simple Stain MAX-PO, Nichirei, Tokyo, Japan) at room temperature for 1 h. Sections were then stained with 3.3′-diaminobenzidine tetrahydrochloride and counter-stained with hematoxylin.

### Microscopic evaluation

The expression of each molecular marker was determined by IHC by comparing relative strength of expression with the adjacent normal proximal tubules as an internal control on the same slide. We defined two qualitative grades for specimens according to the intensity of staining as (i) “high-expression” with a tumor staining intensity equal or stronger than that of normal proximal tubules; and ii) “low-expression” as lower intensity than that of the proximal tubules, or completely absent staining. These semi-quantitative IHC comparisons offer a non-ambiguous evaluation for tumor expression of these molecules. Grading of biomarker expression with IHC was performed by two independent researchers (R.K., T.C.), who had no information on the clinical parameters.

### Risk stratification based on multiple biomarker expression for cancer-specific survivals in renal cell carcinoma

In addition, referring to the hazard values of univariate Cox proportional hazards model analysis, scoring assignments for developing a prognostic model with multiple biomarkers were performed by weighting the contribution score to prognosis for each biomarker as follows: ARL4C, 11 points; ECT2, 3 points; SOD2, 4 points, and STEAP3 10 points (Table 1). According to these weighted scores, we calculated the risk score for each patient and distributed the patients into two different groups, and searched for a meaningful significant cut-off point for the prediction of prognosis.

**Table 1:** Prognostic genes for cancer-specific survival in 97 renal cell carcinomas by univariate analyses

	Univariate		Assigned points
	HR (95% CI)	*p* value†	
ARL4C expression (high vs low)	10.57 (3.62–30.89)	< 0.001	11
ECT2 expression (high vs low)	3.06 (1.11–8.39)	0.030	3
SOD2 expression (high vs low)	3.98 (1.43–11.07)	0.008	4
STEAP3 expression (high vs low)	10.08 (3.25–31.25)	< 0.001	10

†Cox proportional hazards regression models

*HR* hazard ratio, *CI* confidence interval

### Statistical analysis

The primary endpoint of this study was cancer-specific survival (CSS). CSS was defined as the period from the point of diagnosis to the point of death as the result of RCC. Analyzed clinicopathological factors included age, gender, histological type, T classification, vascular involvement, pathological grade, metastasis at diagnosis, and qualitative grade for the immunohistochemical findings. CSS was estimated using the Kaplan-Meier method and compared by log-rank and chi-square tests. The univariate and multivariate Cox proportional hazards regression models were used to evaluate independent prognostic effects of the selected variables with a 95% confidence interval. CSS intervals were used as the indicator for the hazard ratio (HR). A risk model for CSS was developed by calculating the risk score using the value of relative risk from the univariate Cox proportional hazard model. All statistical analyses were performed using SPSS 22.0 (IBM Inc., Chicago, IL). All tests for statistical significance were two-sided. A *p* value < 0.05 was considered statistically significant.

## Results

### Prognostic ability of ARL4C and the other biomarkers for cancer-specific survivals in renal cell carcinoma

**Fig. 1:**
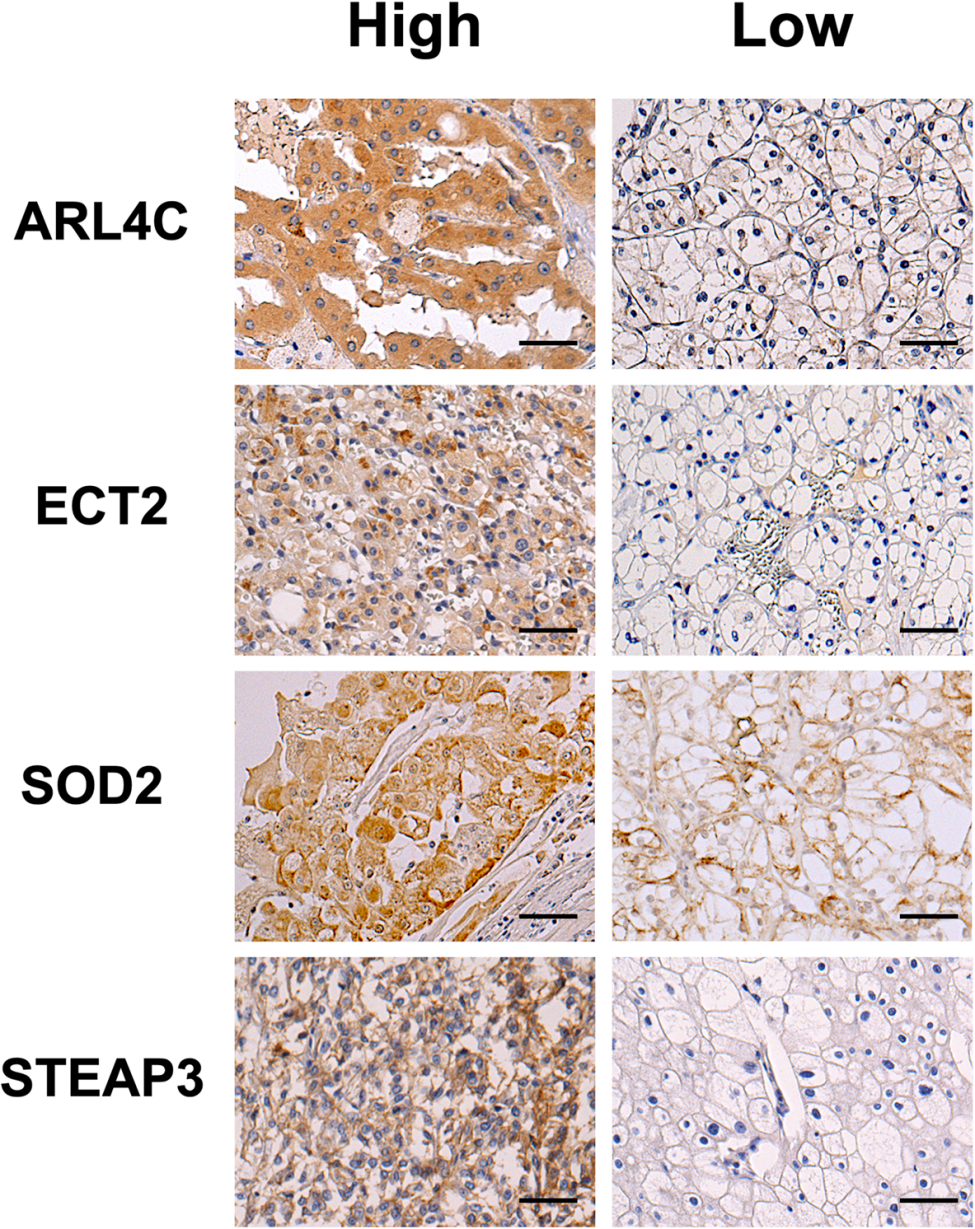
Representative immunohistochemical images in samples from primary tumor tissues of patients with renal cell carcinoma. Expression level of each biomarker is defined as follows; “high-expression” represents staining intensity in tumor cells equal to or higher than that of normal proximal renal tubules; “low-expression” is define as either a negative reaction or lower positive staining than that of proximal tubules. Scale bars indicate 50 μm

**Fig. 2:**
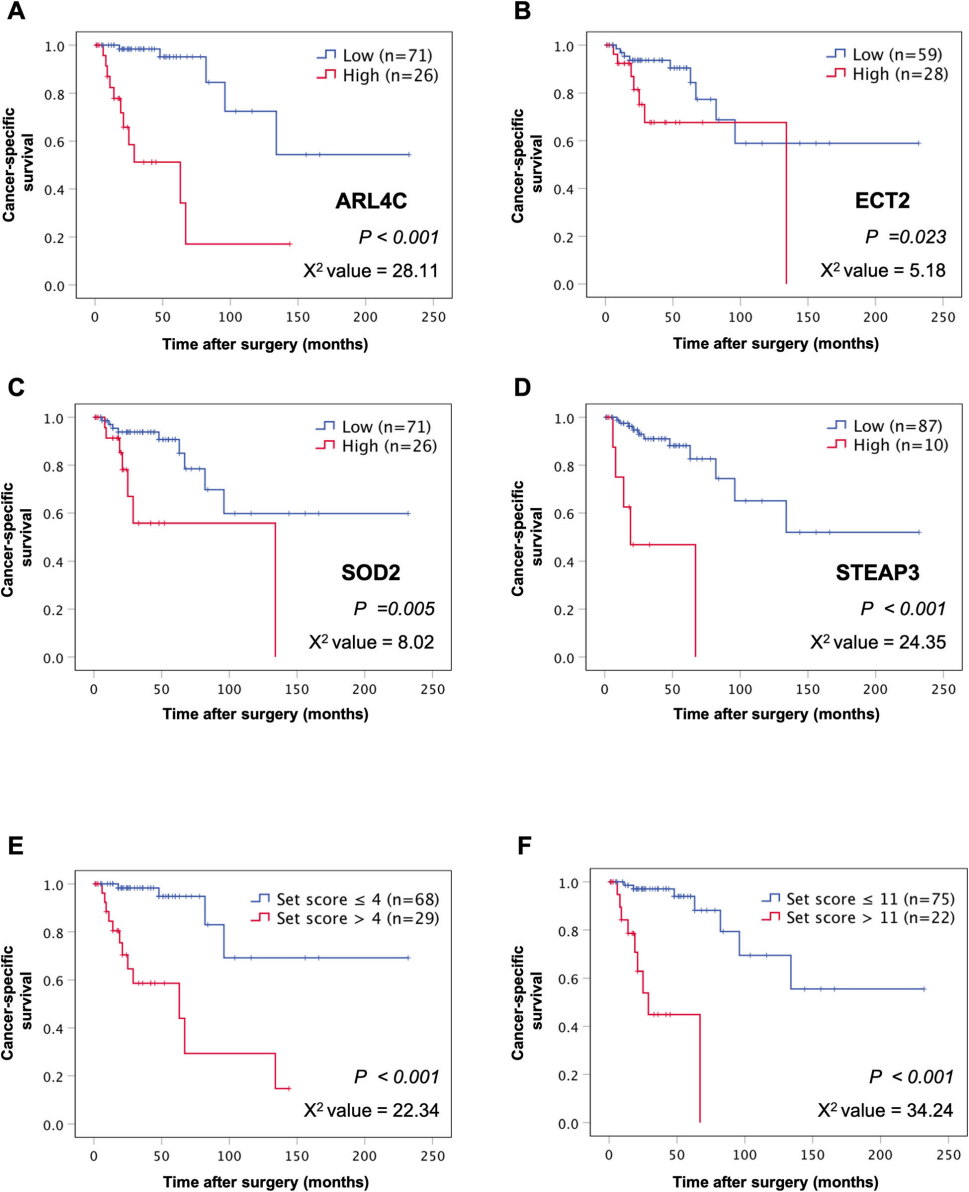
Cancer-specific survival (CSS) rates of the groups categorized by expression levels of **a** ARL4C, **b** ECT2, **c** SOD2, and **d** STEAP3 in a cohort of 97 patients with RCC. The high-expression groups were associated with significantly shorter CSS than the low-expression groups for each individual biomarker (log rank test, **a** *p* < 0.001, chi-square value = 28.11, **b** *p* = 0.023, chi-square value = 5.18, **c** *p* = 0.005, chi-square value = 8.02, **d** *p* < 0.001, chi-square value = 24.35). **e** The patients were divided into high- and low-risk scored groups under our model of risk stratification by setting the cut-off value to 4 points; high- (> 4) risk scored groups had significantly shorter survival than low- (≤ 4) risk scored groups (**e** log-rank test, *p* < 0.001, chi-square value =22.34). The latter had better prognosis without disease-specific death. **f** The cut-off value was shifted to 11 points; the high- (> 11) risk scored group now encompasses patients with a worse prognoses and likely demise within 5 years (**f** log-rank test, *p* < 0.001, chi-square value = 34.24)

Representative cases of high and low expression of each molecular biomarker in pathological specimens are shown in Fig. 1. Expression levels were categorized as high for ARL4C in 26 cases (26.8%), for ECT2 in 28 (28.9%), SOD2 in 26 (26.8%), and STEAP3 in 10 (10.3%). Survival curves were constructed to evaluate the association of each biomarker with the prognosis of RCC. Shorter CSS was seen in cases with stronger expression of ARL4C, ECT2, SOD2, and STEAP3. The 5-year CSS of high and low ARL4C groups was respectively 51.2% and 95.1% with a high chi-square value (log-rank test *p* < 0.001, Chi-square value = 28.11; Fig. 2a). The 5-year CSS was 67.7% and 84.4% for the high and low ECT2 groups, respectively (log-rank test *p* =0.023, chi-square value = 5.18; Fig. 2b), 55.8% and 85.0% for the SOD2 groups (log-rank test *p* = 0.005, chi-square value = 8.02; Fig. 2c), and 46.9% and 82.6% for STEAP3 (log-rank test *p* < 0.001, chi-square value = 24.35; Fig. 2d).

### Risk stratification predicts longer survival and likely deceased cases in the whole cohort of renal cell carcinoma patients

Using univariate Cox proportional hazards analysis, T3/4, histological grade 3, necessity of radical nephrectomy, presence of metastasis at diagnosis, and high expression of all four biomarkers were found to be factors predicting worse prognosis. Multivariate Cox proportional hazards models indicated that the presence of metastasis at diagnosis (HR = 9.17, 95% CI = 1.45-57.83, *p* = 0.018), T3/4 (HR = 12.00, 95% CI = 1.50–96.30, *p* = 0.019) and high ARL4C expression (HR = 10.98, 95% CI = 1.77–68.02, *p* = 0.010) were significant risk factors for worse CSS (Table 2).

**Table 2:** Prognostic evaluation of clinicopathological parameters for cancer-specific survival in 97 renal cell carcinomas by univariate and multivariate analyses

		Univariate		Multivariate	
	n	HR (95% CI)	*p* value†	HR (95% CI)	*p* value†
Sex (male/female)	77/20	0.85 (0.24–3.02)	0.807		
Age, years (> 62 vs ≤ 62)	52/45	0.75 (0.27–2.04)	0.569		
Histological subtype (non-clear vs clear)	21/76	1.30 (0.36–4.75)	0.690		
T classification (3,4 vs 1,2)	25/72	8.76 (2.90–26.42)	< 0.001	**12.00** **(1.50–96.30)**	**0.019**
Vascular invasion (yes vs no)	67/30	36.65 (0.41–3276)	0.116		
Pathological grade (3 vs 1,2)	18/79	12.83 (3.93–41.91)	< 0.001	2.13 (0.42–10.70)	0.358
Nephrectomy (radical vs partial)	51/46	10.27 (1.32–80.06)	0.026	2.24 (0.24–20.94)	0.480
Metastasis at diagnosis (yes vs no)	27/70	23.31 (5.23–103.8)	< 0.001	**9.17** **(1.45–57.83)**	**0.018**
ARL4C expression (high vs low)	26/71	10.57 (3.62–30.89)	< 0.001	**10.98** **(1.77–68.02)**	**0.010**
ECT2 expression (high vs low)	28/69	3.06 (1.11–8.39)	0.030	1.14 (0.06-20.67)	0.928
SOD2 expression (high vs low)	26/71	3.98 (1.43–11.07)	0.008	4.01 (0.24–68.31)	0.337
STEAP3 expression (high vs low)	10/87	10.08 (3.25–31.25)	< 0.001	0.16 (0.02–1.51)	0.109

†Cox proportional hazards regression models

*HR* hazard ratio, *CI* confidence interval

According to the scores contributing to the prediction of prognosis of RCC patients, which were based on hazard risk values of the 4 individual biomarkers in univariate Cox regression analysis, we stratified the patients into two different groups with meaningful cut-off points. By setting the cut-off score to 4 points, meaning only positivity for each SOD2, ECT2 or nothing, 97 patients were divided into high- (> 4) or low- (≤ 4) scored groups. The latter group of 68 cases experienced better survival, without any deaths 8 years after the primary therapeutic procedure of nephrectomy. This remained stably at 69.1% even after more than 10 years (log-rank test *p* < 0.001, chi-square value = 22.34, Fig. 2e). When the cut-off score was set at > 11 points, meaning positivity for ARL4C and additionally another biomarker, none of the 22 patients in this high-risk scored group were alive 5 years after primary nephrectomy (log rank test *p* < 0.001, chi-square value = 34.24, Fig.2f). In comparison to ARL4C as the most prognostic potent single biomarker, risk stratification at > 11 points predicted a worse prognosis.

### Association of four gene products with cancer-specific survivals in metastatic renal cell carcinomas and prognostic performance of multiple biomarker models

**Fig. 3:**
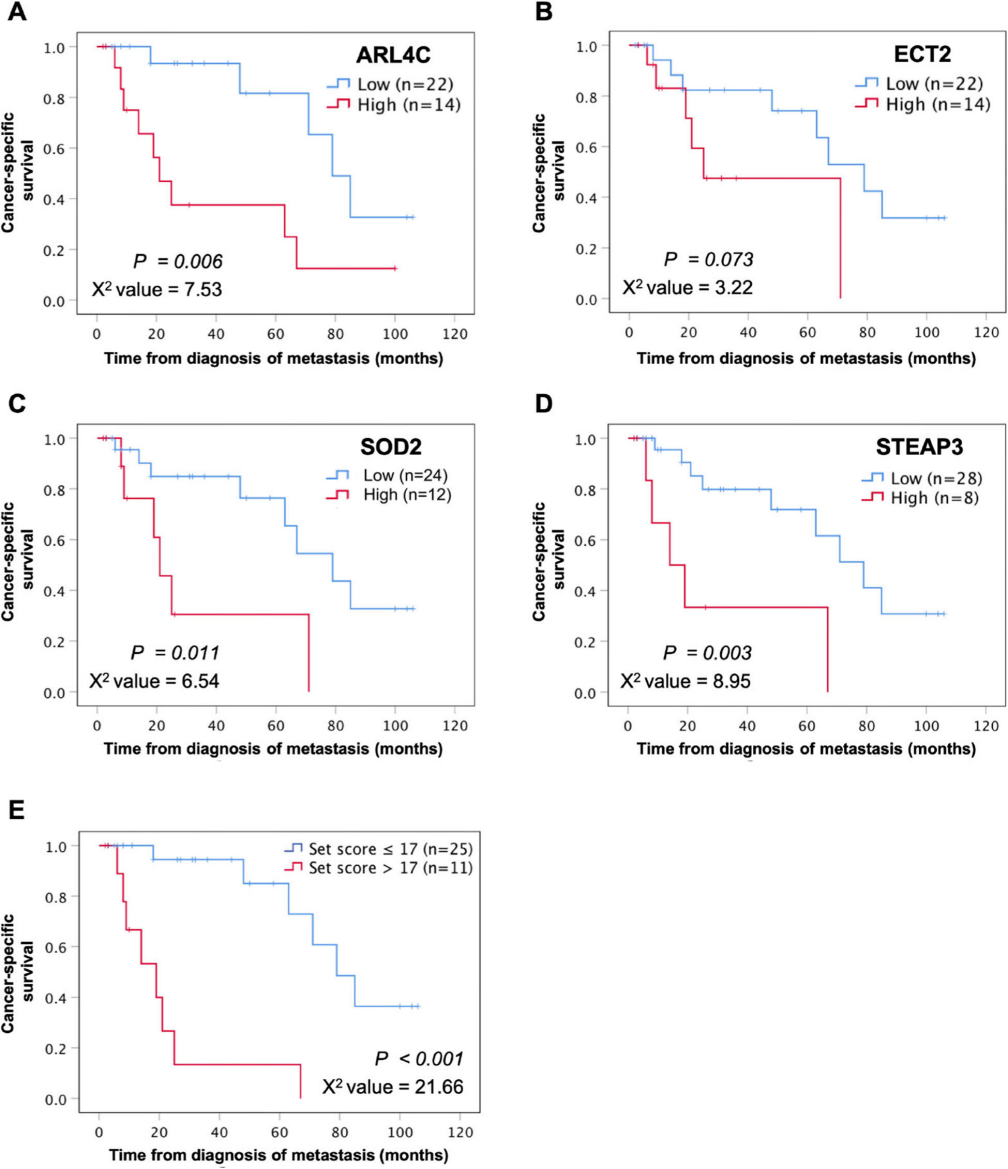
Cancer-specific survival rates of the groups categorized by expression levels of **a** ARL4C, **b** ECT2, **c** SOD2, and **d** STEAP3 in a cohort of 36 patients with metastatic RCC. Shorter survival from the time of diagnosis of metastasis is seen in cases with higher expression levels of ARL4C (log-rank test, **a** *p* = 0.006, chi-square value = 7.53), SOD2 (log-rank test, **c** *p* = 0.011, chi-square value = 6.54), and STEAP3 (log-rank test, **d** *p* = 0.003, chi-square value = 8.95). **e** The patients were divided into high- and low-risk scored groups under our model of risk stratification by setting the cut-off value to 17 points; the high- (> 17) risk score predicted likely demise within 2 years more accurately than each individual biomarker (log-rank test, **e** *p* < 0.001, chi-square value = 21.66)

We analyzed 36 cases of metastatic RCCs including 27 cases of metastasis at the initial diagnosis and 9 of distant occurrence after first surgery in order to evaluate the prognostic capacity of our risk stratification model and to compare its performance with that of each individual biomarker. Shorter CSS from the time of diagnosis of metastasis was significantly associated with stronger expression of ARL4C, SOD2, and STEAP3. The 2-year CSS from diagnosis of metastasis in high and low ARL4C groups was 46.9% and 93.3%, respectively (log-rank test *p* = 0.006, chi-square value = 7.53, Fig. 3a). These values were 59.3% and 82.4% for ECT2 (log-rank test *p* = 0.073, chi-square value = 3.22, Fig. 3b), 45.7% and 84.8% for SOD2 (log-rank test *p* = 0.011, chi-square value = 6.54; Fig. 3c), and 46.9% and 82.6% for STEAP3 (log-rank test *p* = 0.003, chi-square value = 8.95, Fig, 3d), respectively. According to multivariate Cox proportional hazards models, the presence of metastasis at diagnosis (HR = 6.26, 95% CI = 1.19-33.00, *p* = 0.031) and high SOD2 expression (HR = 4.97, 95% CI = 1.12–22.02, *p* =0.035) were significantly associated with shorter CSS intervals in 36 metastatic RCC patients (Table 3). Compared to SOD2, our model for risk stratification performed well, especially for predicting early deaths when the cut-off score was increased to > 17 points; i.e., positivity of ARL4C and additionally STEAP3 or > 2 other biomarkers. With this cut-off, the 2-year CSS from diagnosis of metastasis in high- (> 17) and low- (≤ 17) risk-scored groups was 26.7% and 94.4%, respectively (log-rank test *p* < 0.001, chi-square value = 21.66, Fig. 3e). The high-scored group had progressively lower survival rates of 26.7–13.3% (Fig. 3e) than those of 45.7–30.5% in the high-SOD2 group (Fig. 3c), at 2–5 years’ follow-up, respectively.

**Table 3:** Prognostic evaluation of clinicopathological parameters for cancer-specific survival in 36 cases of metastatic renal cell carcinomas by univariate and multivariate analyses

		Univariate		Multivariate	
	n	HR (95% CI)	*p* value†	HR (95% CI)	*p* value†
Sex (male/female)	30/6	2.56 (0.68–9.76)	0.164		
Age, years (> 62 vs ≤ 62)	18/18	0.52 (0.17–1.57)	0.247		
Histological subtype (non-clear vs clear)	5/31	0.65 (0.08–5.25)	0.652		
T classification (3,4 vs 1,2)	20/16	2.83 (0.89–9.00)	0.077		
Vascular invasion (yes vs no)	34/2	25.44 (0.02–44141)	0.395		
Pathological grade (3 vs 1,2)	13/23	4.82 (1.36–17.06)	0.015	1.24 (0.25–6.17)	0.792
Metastasis at diagnosis (yes vs no)	27/9	5.41 (1.16–25.26)	0.032	**6.26** **(1.19–33.00)**	**0.031**
Molecular targeting therapy (yes vs no)	25/11	1.33 (0.44–4.00)	0.617		
ARL4C expression (high vs low)	15/21	4.20 (1.39–12.72)	0.011	2.37 (0.52–10.51)	0.273
ECT2 expression (high vs low)	15/21	2.85 (0.87–9.34)	0.084		
SOD2 expression (high vs low)	12/24	4.07 (1.28–12.94)	0.018	**4.97** **(1.12–22.02)**	**0.035**
STEAP3 expression (high vs low)	8/28	5.16 (1.56–17.08)	0.007	2.19 (0.45–9.970)	0.343

†Cox proportional hazards regression models

*HR* hazard ratio, *CI* confidence interval

### Risk stratification predicts prognosis of metastatic renal cell carcinoma patients treated with molecular targeting therapies

**Fig. 4:**
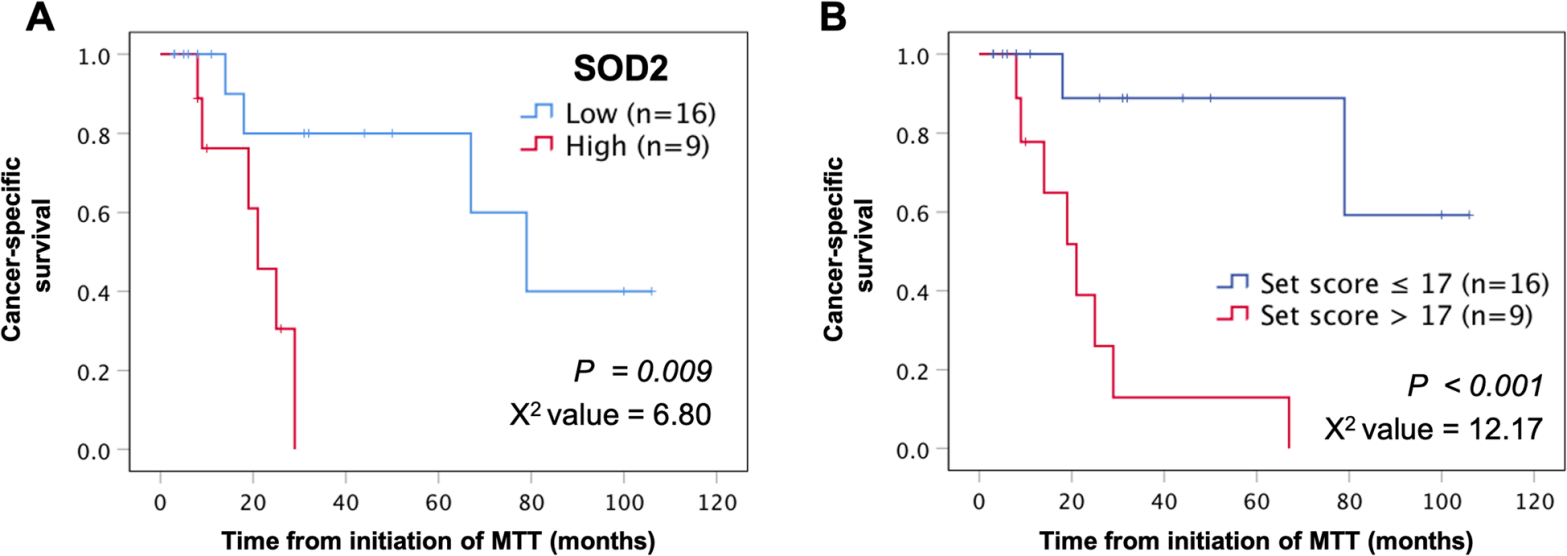
Cancer-specific survival (CSS) curves for 25 metastatic RCC treated with molecular targeting therapy (MTT) under the evaluation of **a** high or low SOD2 expression, and of **b** high- (> 17) or low- (≤ 17) risk scoring in our model of risk stratification. In this sub-cohort, CSS was defined as the period from the point of initiation of MTT to the time of RCC-specific death. Shorter CSS was observed in the high SOD2 expression group (**a** log-rank test, *p* = 0.009, chi-square value = 6.80) and highest-risk (> 17) scored groups (**b** log-rank test, *p* < 0.001, chi-square value = 12.17). Compared to the highest-risk scored group, high SOD2 expression could accurately predict those patients likely to be dead within 30 months of initiation of MTT

In addition, 25 cases treated with molecular targeted therapies (MTT) were analyzed in order to evaluate therapeutic efficacy. According to this additionally sub-group analysis, the implementation of current MTT did not improve CSS in cases displaying high SOD2 expression (log-rank test *p* = 0.009, chi-square value = 6.80, Fig. 4a), or the highest (> 17 points) risk-scored patients in our model of risk stratification (log-rank test *p* < 0.001, chi-square value = 12.17; Fig. 4b). In contrast to our model of risk stratification, high SOD2 expression was better able to identify patients with the worst prognosis in that all 9 patients with high SOD2 expression had died by 30 months from initiation of MTT.

## Discussion

In this study, we identified two clinical meaningful findings by developing a risk-stratified model based on the expression profiles of four biomarkers and by validating its prognostic significance in our cohort. First, our risk model could predict the prognosis of patients with RCC more accurately and identify cases requiring closer follow-up and medical management. Second, the current molecular targeting therapies (MTT) are insufficient to improve survival of metastatic RCC patients in the high-risk group of our model, especially in those with high SOD2 expression.

Higher expression levels by IHC for each of the four biomarkers in primary RCC tissue were significantly associated with poor prognosis in the overall cohort of 97 cases including all stages of RCC. Of these 4 biomarkers, ARL4C is a transcriptional factor induced by cooperative signaling of Wnt/β-catenin and EGF/Ras-MAPK signaling activation, which promotes proliferation, migration, and invasion of cancer cells [16]. In previous studies, high ARL4C expression was associated with poor prognosis in some carcinomas [16–18]. Another of the four biomarkers, ECT2, is a guanine exchange factor for the Rho family of GTPases that has been associated with the regulation of cell cycle progression and cytokinesis. The dysregulation of ECT2 leads to malignant transformation and enhances metastatic ability through upregulating the epithelial mesenchymal transition process [19]. The third biomarker, SOD2, is a primary mitochondrial antioxidant reducing reactive oxygen species (ROS), and which can prevent cell death and promote epithelial to mesenchymal transition and/or cancer cell migration [9, 20]. Finally, STEAP3 is a member of the iron regulatory protein family and is a ferrireductase which reduces ferric iron to ferrous iron in endosomes. It has been reported that STEAP3 overexpression facilitates iron uptake, maintains iron storage in cancer cells and supports cancer cell proliferation under hypoferric conditions [21, 22].

ARL4C was shown to be the most prognostic single biomarker among four markers studied in multivariate analysis, but our risk-stratified model with modified cut-off value for the risk score showed better accuracy than ARL4C prediction alone. When the cut-off point was set to 4, low- (≤ 4) scored patients, i.e., positive for SOD2, ECT2, or nothing, showed better survival rates and none was deceased 8 years after primary nephrectomy. In cases without expression of any biomarkers or with only expression of SOD2 or ECT2 alone, close follow-up may not be necessary except in metastatic cases. Conversely, by setting the cut-off point to 11, the high-(> 11) scored group, i.e., positive for ARL4C and additionally another biomarker, identified patients likely to be dead within 5 years. These results indicate that ARL4C may contributes to more aggressive behavior of the tumor including a possibility of metastasis, as demonstrated that high expression of ARL4C in lung, liver, and colorectal cancers was an independent indicator of relapse [23, 24]. The other three biomarkers may promote the progression of metastatic tumors. These findings can offer us useful information on the selection of cases for optimal medical management. Thus, follow-up of RCC cases with abundant ARL4C and additionally another biomarker should be more closely controlled with more frequently follow-up.

In 36 patients with metastatic RCC, high- (> 17) scored patients in our risk model, meaning positivity of ARL4C or STEAP3 and additionally more than two other biomarkers, always had worse survival and death at 2 to 5 years after diagnosis of metastasis. In our model, among 25 patients who received MTT, both the high-(> 17) scored group and the high SOD2 group had a very poor prognosis after initiation of MTT. In addition, the latter SOD2 group predicted imminent death more accurately. These findings indicate that each of the four molecules coordinately accelerated the progression of metastatic cancers through individually different pathways, but interestingly the most important indicator determining whether long-term survival can be achieved by the current MTT was simply the SOD2 expression level. Therefore, we believe that novel and innovative therapeutic strategies are required to achieve better prognosis in patients with metastatic RCCs showing high SOD2 expression. The one of mechanisms of MTT including inhibitors of tyrosine kinase and mTOR kinase is based on nutrition-starvation via inhibition of angiogenesis and/or interfering the cellular signaling of the aberrant proliferation activity of tumor cells [25, 26]. However, cancer cells expressing highly SOD2 can survive in a nutritionally depleted environment via preserving mitochondrial function and preventing ROS accumulation [27]. This helps to maintain the dormant character in RCC development [8, 9]. It has been previously demonstrated that inhibitors targeting mitochondrial oxidative phosphorylation such as biguanides induce death of RCC cells and may become therapeutic options to overcome the disastrous consequences of SOD2-abundance in RCC cells [9]. Furthermore, the antitumor effect of biguanides like metformin in patients with diabetes has been demonstrated in several carcinomas [28–32]. In order to target tumor cell mitochondria, applying biguanides to replacement therapy or drug repositioning should be proposed for patients with SOD2-abundant RCC for maintenance phase treatments after primary surgery debulking numbers of RCC cells.

Important limitations exist in the present study beginning with the sample being drawn from a population of patients at a single, small institute. The sample size, heterogeneity of the analyzed patients and lack of external validation cohort may prevent generalization of the study results to patients at the other institutions. However, even under such small and heterogenic cohort, the present analysis has indicated enough statistical differences, and so we cannot deny that these announced novel biomarkers are meaningful in prognostic and therapeutic prediction of RCC patients. Secondly, it has not been verified yet whether our risk-stratified model can predict the prognosis of the patients who received the current ICI treatment. Our cohort has included a number of patients who previously treated before application of the current ICI therapy. Under the current prosperity of ICI therapeutics, the opportunities receiving a first-line MTT are declining. However, MTT has been still required as an alternative to ICI, in the patients who cannot receive or tolerate ICI or who are refractory to ICI. Therefore, it has been still essential to predict the prognosis of patients undergoing MTT. What remains unresolved is whether the assessment is reproducible by other investigators. However, immunohistochemical evaluation is widely available at almost all hospitals and can allow molecular expression levels to be evaluated by qualitative grading in tumor cells by comparing the staining intensity of RCC tissues with adjacent normal proximal tubules.

We propose a risk stratification model constructed by four novel biomarkers (ARL4C, ECT2, STEAP3, SOD2) that can be used to predict CSS in RCC patients. The risk stratification accurately predicts CSS in both whole RCC patients and metastatic RCC sub-group. Although our risk model showed enough prognostic prediction, more accurate predictions might be performed, if the risk model had included BIRC5 and BUB1 which had been well-established prognostic function for RCC patients [12, 13]. We hope further prospective studies with more samples from different medical centers will be able to validate our prognostic signature and confirm its clinical utility.

## Conclusions

This study demonstrated that a risk-stratified model based on four survival-related biomarkers could contribute to the clinical implications of prognostic prediction and generate useful information for considering follow-up and therapeutic strategies for patients with primary and metastatic RCC.

## Data Availability

The analyzed datasets generated during the study are possibly available from the corresponding author on reasonable request.

## Abbreviations

ARL4C: ADP-ribosylation factor-like 4C
ECT2: Epithelial cell transforming 2
ICI: Immune checkpoint inhibitor
IHC: Immunohistochemistry
MTT: Molecular targeting therapy
CSS: Cancer-specific survival
RCC: Renal cell carcinoma
ROS: Reactive oxygen species
SOD2: Superoxide dismutase 2
STEAP3: STEAP3 metalloreductase
